# Genetics of sleep medication purchases suggests causality from sleep problems to psychiatric traits

**DOI:** 10.1093/sleep/zsad279

**Published:** 2023-11-20

**Authors:** Martin Broberg, Viola Helaakoski, Tuomo Kiiskinen, Tiina Paunio, Samuel E Jones, Nina Mars, Jacqueline M Lane, FinnGen, Richa Saxena, Hanna M Ollila

**Affiliations:** Institute for Molecular Medicine Finland (FIMM), University of Helsinki, Helsinki, Finland; Institute for Molecular Medicine Finland (FIMM), University of Helsinki, Helsinki, Finland; Institute for Molecular Medicine Finland (FIMM), University of Helsinki, Helsinki, Finland; Genomics and Biomarkers Unit, Finnish Institute for Health and Welfare, Helsinki, Finland; Department of Psychiatry and SleepWell Research Program, Faculty of Medicine, University of Helsinki and Helsinki University Central Hospital, Helsinki, Finland; Institute for Molecular Medicine Finland (FIMM), University of Helsinki, Helsinki, Finland; Institute for Molecular Medicine Finland (FIMM), University of Helsinki, Helsinki, Finland; Division of Sleep and Circadian Disorders, Brigham and Women’s Hospital and Harvard Medical School, Boston, MA, USA; Division of Sleep and Circadian Disorders, Brigham and Women’s Hospital and Harvard Medical School, Boston, MA, USA; Center for Genomic Medicine, Massachusetts General Hospital, Boston, MA, USA; Program in Medical and Population Genetics, Broad Institute, Cambridge, MA, USA and; Department of Anesthesia, Critical Care and Pain Medicine, Massachusetts General Hospital and Harvard Medical School, Boston, MA, USA; Institute for Molecular Medicine Finland (FIMM), University of Helsinki, Helsinki, Finland; Center for Genomic Medicine, Massachusetts General Hospital, Boston, MA, USA; Program in Medical and Population Genetics, Broad Institute, Cambridge, MA, USA and; Department of Anesthesia, Critical Care and Pain Medicine, Massachusetts General Hospital and Harvard Medical School, Boston, MA, USA

**Keywords:** genetics, sleep hygiene, insomnia

## Abstract

**Study Objectives:**

Over 10% of the population in Europe and in the United States use sleep medication to manage sleep problems. Our objective was to elucidate genetic risk factors and clinical correlates that contribute to sleep medication purchase and estimate the comorbid impact of sleep problems.

**Methods:**

We performed epidemiological analysis for psychiatric diagnoses, and genetic association studies of sleep medication purchase in 797 714 individuals from FinnGen Release 7 (*N* = 311 892) and from the UK Biobank (*N* = 485 822). Post-association analyses included genetic correlation, co-localization, Mendelian randomization (MR), and polygenic risk estimation.

**Results:**

In a GWAS we identified 27 genetic loci significantly associated with sleep medication, located in genes associated with sleep; *AUTS2*, *CACNA1C*, *MEIS1*, *KIRREL3*, *PAX8*, *GABRA2*, psychiatric traits; *CACNA1C*, *HIST1H2BD*, *NUDT12. TOPAZ1* and *TSNARE1.* Co-localization and expression analysis emphasized effects on the *KPNA2*, *GABRA2*, and *CACNA1C* expression in the brain. Sleep medications use was epidemiologically related to psychiatric traits in FinnGen (OR [95% (CI)] = 3.86 [3.78 to 3.94], *p *< 2 × 10^−16^), and the association was accentuated by genetic correlation and MR; depression (*r*g = 0.55 (0.027), *p *= 2.86 × 10^−89^, *p* MR = 4.5 × 10^−5^), schizophrenia (*r*_g_ = 0.25 (0.026), *p *= 2.52 × 10^−21^, *p* MR = 2 × 10^−4^), and anxiety (*r*g = 0.44 (0.047), *p *= 2.88 × 10^−27^, *p* MR = 8.6 × 10^−12^).

**Conclusions:**

These results demonstrate the genetics behind sleep problems and the association between sleep problems and psychiatric traits. Our results highlight the scientific basis for sleep management in treating the impact of psychiatric diseases.

## Graphical Abstract

**Graphical Abstract:**
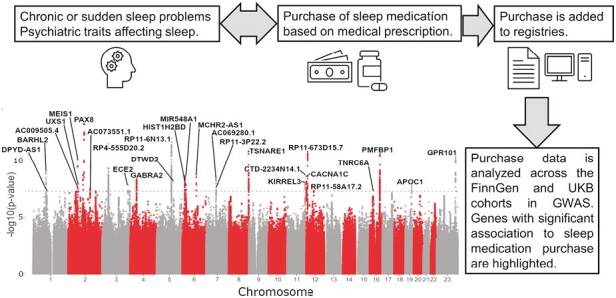

**Statement of Significance**

This study describes a novel approach to exploring the genetics of sleep problems, primarily insomnia, using non-benzodiazepine purchase registry data for a genome-wide association study. For this approach, we utilize the FinnGen research project Release 7 (*N* = 311 892) and the UK Biobank (*N* = 485 822). The results are followed up with Mendelian randomization, polygenic risk analysis, and genetic correlation analysis to provide a wider understanding of how the results relate to insomnia and psychiatric trait comorbidities. Furthermore, we perform eQTL and co-localization analysis to further clarify the potential impact of the 27 lead SNPs detected in the main analysis. These results demonstrate the genetics behind sleep problems and their association with psychiatric traits, highlighting sleep management in treating psychiatric diseases.

## Introduction

Sleep disorders are common in the general population with approximately a 20%–30% lifetime prevalence [1, 2]. Occasional sleep problems are normal and can constitute occasional insomnia or jet lag or overall poor sleep quality. Such sleep problems can cause daytime sleepiness, sleep fragmentation, and originate also from short habitual sleep duration. However, prolonged or severe sleep problems such as frequent insomnia, excessive daytime sleepiness, sleep apnea, restless legs syndrome, circadian rhythm disorders, or any sleep problems that affect daily functioning require treatment [3, 4]. Sleep problems and are usually treated with either non-pharmaceutical tools such as improving sleep hygiene or cognitive behavioral therapy, or alternatively or in combination of sleep medication which comprise benzodiazepine related drugs (ICD 10/ATC class N05CF) introduced in the 1980s for insomnia treatment [5]. These so-called sleep medication or non-benzodiazepine drugs (Zopiclone; ATC code N05CF01, Zolpidem; ATC code N05CF02, Zaleplon; ATC code N05CF03 and Eszopiclone; ATC code N05CF04) are now the most prescribed sleep medication globally, and estimations put the general use of sleep promoting drugs to about 5%–30% in the general elderly population (over 65 years of age) [5, 6]. The primary indication of sleep medication for use in Finland is sleep problems. The use of sleep medication is more common in women compared to men [7].

All sleep medications in the sleep medications group bind gamma-aminobutyric acid receptors. Zopiclone is a hypnosedative GABA A receptor inhibitor, binding both the α1 and α2 parts of the receptor, reducing GABA based synapses. Zolpidem is a more selective inhibitor than Zopiclone, targeting the ω1 site of the GABA A receptor [8]. Zolpidem has been used at times to temporarily treat neurological disorders such as strokes, Parkinson’s disease, and dementia [5, 8]. Zaleplon also binds the ω1 site of the GABA receptor [9]. The primary benefit of Zaleplon is significantly shortened sleep latency.

Earlier work by us and others have shown that there is a significant link between sleep problems and mental health conditions such as alcohol use disorder, depression, anxiety, and schizophrenia [10, 11]. Recent studies have reported significant associations between decline in cognitive abilities and the purchases of sleep medications, confounded by disorders such as depression and insomnia that are associated with age-related cognitive decline and dementia [12, 13]. Previous GWASs have assessed the genetic component and heritability of sleep problems; for example in daytime sleepiness heritability has been estimated to 0.1–0.2, short sleep duration is approximately 0.07 [14, 15], and insomnia at 0.07 [16, 17]. Furthermore, GWASs of sleep quality have also demonstrated significant heritability of sleep patterns based on accelerometer data at 0.1 [18] and genetic correlation between insomnia (self-reported) and depression (*r*g = 0.5), neuroticism (*r*g = 0.5), and negative correlation to overall well-being (*r*g = −0.4) [17]. However, to date there are no population-wide GWASs on the use of sleep medication and their genetic links to psychiatric traits.

## Materials and Methods

### Study cohorts and phenotypes

#### FinnGen.

FinnGen is a large-scale research study that aims to genotype 500 000 Finnish participants recruited from hospitals, as well as prospective and retrospective epidemiological and disease-based cohorts. These data are combined with longitudinal registries that record phenotypes and health events (including ICD-based diagnosis) over the entire lifespan including the National Hospital Discharge Registry (inpatient and outpatient), Causes of Death Registry, the National Infectious Diseases Registry, Cancer Registry, Primary Health Care Registry (outpatient), and Medication Reimbursement Registry.

This study used data from FinnGen Data Freeze 7, which comprises 311 892 individuals. For the purposes of our research, we used registered sleep medication purchases (which constitute a prescription-only medication in Finland) dating from 1995. The primary indication for sleep medication prescription in Finland is sleep problems, and of these, non-benzodiazepine drugs are used for insomnia treatment [19].

### FinnGen ethics statement

Patients and control participants in FinnGen provided informed consent for biobank research, based on the Finnish Biobank Act. Alternatively, separate research cohorts, collected before the Finnish Biobank Act came into effect (in September 2013) and before start of FinnGen (August 2017), were collected based on study-specific consents and later transferred to the Finnish biobanks after approval by Fimea (Finnish Medicines Agency), the National Supervisory Authority for Welfare and Health. Recruitment protocols followed the biobank protocols approved by Fimea. The Coordinating Ethics Committee of the Hospital District of Helsinki and Uusimaa (HUS) statement number for the FinnGen study is Nr HUS/990/2017.

The FinnGen study is approved by the Finnish Institute for Health and Welfare (permit numbers: THL/2031/6.02.00/2017, THL/1101/5.05.00/2017, THL/341/6.02.00/2018, THL/2222/6.02.00/2018, THL/283/6.02.00/2019, THL/1721/5.05.00/2019, and THL/1524/5.05.00/2020), Digital and population data service agency (permit numbers: VRK43431/2017-3, VRK/6909/2018-3, and VRK/4415/2019-3), the Social Insurance Institution (permit numbers: KELA 58/522/2017, KELA 131/522/2018, KELA 70/522/2019, KELA 98/522/2019, KELA 134/522/2019, KELA 138/522/2019, KELA 2/522/2020, and KELA 16/522/2020), Findata permit numbers THL/2364/14.02/2020, THL/4055/14.06.00/2020, THL/3433/14.06.00/2020, THL/4432/14.06/2020, THL/5189/14.06/2020, THL/5894/14.06.00/2020, THL/6619/14.06.00/2020, THL/209/14.06.00/2021, THL/688/14.06.00/2021, THL/1284/14.06.00/2021, THL/1965/14.06.00/2021, THL/5546/14.02.00/2020, and Statistics Finland (permit numbers: TK-53-1041-17 and TK/143/07.03.00/2020 [earlier TK-53-90-20]).

The Biobank Access Decisions for FinnGen samples and data utilized in FinnGen Data Freeze 7 include: THL Biobank BB2017_55, BB2017_111, BB2018_19, BB_2018_34, BB_2018_67, BB2018_71, BB2019_7, BB2019_8, BB2019_26, and BB2020_1; Finnish Red Cross Blood Service Biobank 7.12.2017; Helsinki Biobank HUS/359/2017; Auria Biobank AB17-5154 and amendment #1 (August 17 2020); Biobank Borealis of Northern Finland_2017_1013; Biobank of Eastern Finland 1186/2018 and amendment 22 §/2020; Finnish Clinical Biobank Tampere MH0004 and amendments (February 21, 2020 and October 06, 2020); Central Finland Biobank 1-2017; and Terveystalo Biobank STB 2018001.

### The UK Biobank

The UK Biobank consists of over 500 000 participants aged between 40 and 69 years, that were assessed between 2006 and 2010 across 22 assessment centers across the United Kingdom. The assessment was based on informed consent and consisted of an interview phase, filling out a touch screen questionnaire, blood, saliva, and urine sampling for analysis, and physical and functional measurements. Longitudinal diagnostic data were provided from health care records (Hospital in-patient and Primary care). This study is based on UKB Application 22627.

We built a similar phenotype to FinnGen in UK Biobank and used the registered events of a sleep medication purchase in the UKB as the phenotype for the GWAS.

### GWAS analysis

To detect the genetic associations with sleep medication purchases we performed GWAS analysis using SAIGE [20] v0.36.3.2 and the FinnGen release 7, separately for participants identified as males (*N* = 135 408), females (*N* = 173 746), as well as for all available participants (*N* = 311 892). We included participants from 1995 onwards, when prescribed medication purchase data became available in Finland. We counted the prescription-based purchase events where participants had been prescribed sleep medications, and then normalized the values using the “rnorm” function in R. The normalized values were used as a quantitative trait in the GWAS. The main parameters used for SAIGE were: covariates: sex, age, age [2], 10 principal components of ancestry and microarray genotyping batch, ratio coefficient of variation cutoff: 0.001, trace coefficient of variation cutoff: 0.0025, minmac: 5, other settings were default for the software. The specific SAIGE GENE analysis type was set to “additive,” and the trait type set to “quantitative.”

As a sensitivity analysis we performed the GWAS using sleep medication purchases as a “binary” trait for analysis, using those who had 1 or more registered purchases as “case” (*N* = 78 790) and those without as “control” (*N* = 233 102). Additionally, we performed a binary analysis using those with 2+ purchases as “case” (*N* = 55 858) and those with 1 or less as “control” (*N* = 256 034).

We followed with a UK Biobank (UKB) GWAS (*N* = 485 822) using the same quantitative trait as for FinnGen (“rnorm” normalized purchase events for participants), but using REGENIE [21] v2.2.4 as analysis software with minMAC: 3 and standard options. Age, sex, age [2], 10 principal components, variant array, and assessment center were used as covariates in the analysis.

The FinnGen and UKB GWAS results were subsequently meta-analyzed using the STDERR method in METAL [22] 2011 version (combined *N* = 794 976).

### Genetic correlation

To understand the overall genetic overlap between sleep medication purchases and relevant traits, genetic correlation was performed using the LDSC software [23] v1.0.1 and the 1000G European phase 3 LD reference panel. We computed the correlation of FinnGen sleep medication purchases against 40 other traits; 15 psychiatric disorder traits, 13 sleep disorder traits, and 10 substance abuse associated traits (Supplementary Table S2). Additionally, the FinnGen sleep medication purchases were correlated against UKB sleep medication purchases. Source studies for the summary statistics and lead SNPs used in the genetic correlation are provided in Supplementary References.

### Mendelian randomization analysis

Based on the genetic correlation, we chose 15 general, psychiatric, sleep, and substance abuse traits for Mendelian randomization (MR) analysis to further understand their causality. These traits included: Parkinson’s disease, Alzheimer’s disease, attention-deficit/hyperactivity disorder (ADHD), post-traumatic stress disorder, alcohol use disorder, weekly alcohol consumption, bipolar disorder, body mass index, diabetes, parental longevity, insomnia, anxiety, depression, neuroticism, schizophrenia, and multisite chronic pain. The MR analysis was performed using the TwoSampleMR [24] R-package. There are multiple methods for MR analysis. The one we focused on in this study is the inverse-variance weighting method. The inverse-variance weighting method produces a weighted regression averaged ratio estimate of the exposure instruments to the outcome to calculate an overall causal estimate. Source studies for the summary statistics and lead SNPs used are provided in Supplementary References.

### Polygenic risk score analysis

To study the genetic overlap between sleep medication purchases as polygenic risk and the further test the significant association with risk factors, we performed a PRS analysis. Using PRS-CS (PRS-CS-auto) [25], we generated PRS based on the GWAS summary statistics for the normalized sleep medication purchases. We used the 1000 Genomes phase 3 European panel as LD reference and the UKB cohort as test panel. Polygenic risk score calculation was performed with PLINK 2 v2.00 [26]. The PRS for sleep medication purchases was z-normalized and then tested in a generalized linear model logistic regression analysis using R v4.2, against the UKB data fields 2050 (frequency of depressed mood in last 2 weeks), 4526 (happiness), 2090 (anxiety-seen doctor), 21062 (anxiety), 1180 (chronotype), 1200 (insomnia), 1980 (worrier/anxious feelings), and 2956 (general pain for ≥3 months), and additionally post-traumatic stress disorder (diagnosis ICD10 F43.1) and depression (diagnosis ICD10 F32, F33) using age, sex, and PCs 1–10 as covariates.

### Expression quantitative trait loci analysis and co-localization analysis

To estimate the impact of the sleep medication purchase-associated SNPs on gene expression, we performed expression quantitative trait loci (eQTL) analysis. We used the eQTL calculator on the GTEx platform [27, 28] for the lead SNPs in the whole cohort analysis, in the following tissues; Brain_Amygdala, Brain_Anterior_cingulate_cortex_BA24, Brain_Caudate_basal_ganglia, Brain_Cerebellar_Hemisphere, Brain_Cerebellum, Brain_Cortex, Brain_Frontal_Cortex_BA9, Brain_Hippocampus, Brain_Hypothalamus, Brain_Nucleus_accumbens_basal_ganglia, Brain_Putamen_basal_ganglia, Brain_Spinal_cord_cervical_c-1, and Brain_Substantia_nigra. We further confirmed the relevance of the eQTL expression and associated brain tissue data with the GWAS summary statistics using the co-localization analysis platform LocusFocus [29]. The LocusFocus platform provides both simple sum and COLOC2 [30] posterior probability results.

## Results

### Demographic analysis of drug purchases using registry data

Given the earlier association between sleep problems and psychiatric traits [10, 16], we examined the demographic characteristics of sleep medication purchasers in both FinnGen and UKB (Supplementary Tables S3–S5). A descriptive analysis showed a higher percentage of females (55.7%) [31] overall in FinnGen and among individuals who used sleep medications (59.5%). Furthermore, 25% of all individuals had at least one prescription of sleep medications in FinnGen and the purchases were more frequent in individuals with a diagnosis for a psychiatric trait (R generalized linear model (glm) regression analysis; *β* = 1.35, SD = 0.011, *p *< 2 × 10^−16^, Supplementary Table S3).

### GWAS identifies 27 lead SNPs for sleep medication purchases

**Figure 1.**
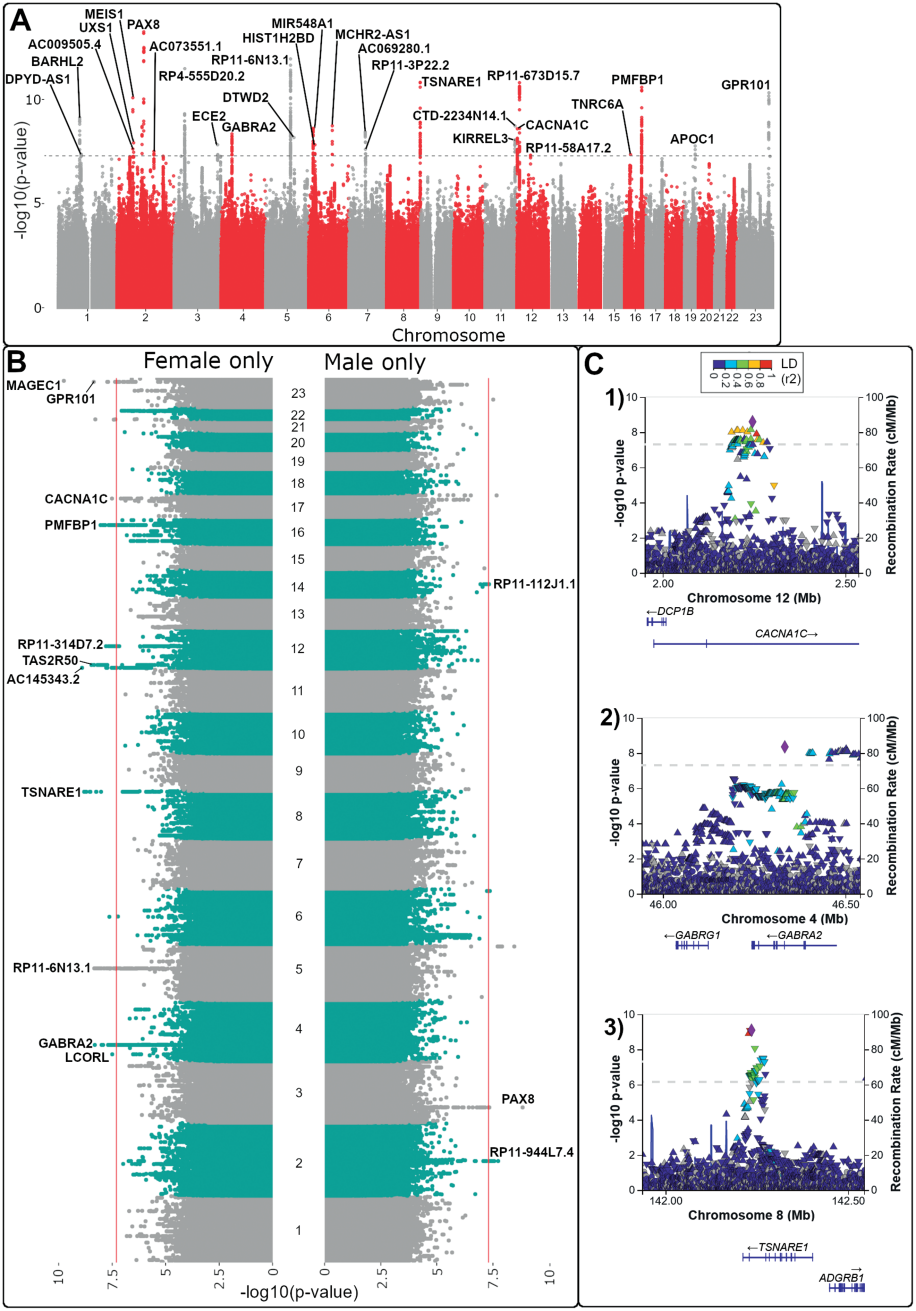
GWAS highlights distinct genetic patterns between males and females associating with sleep medication purchases. (A) Manhattan plot of the FinnGen study used in this investigation. (B) Miami plot of female and male-only subgroups of the total FinnGen R7 study. (C) LocusZoom plots of the lead SNPs rs2370413, rs279829, rs13262595, and rs1190734.

We identified 27 independent genetic loci that associated with sleep medication purchases (Figure 1, A and B, Supplementary Table S6). These were located in the regions of genes connected to sleep (*AUTS2*, *BARHL2*, *ID4*, *KIRREL3*, *MEIS1*, *PAX8*, and *PMFBP1*), genes connected with psychiatric and personality traits (*CACNA1C*, *HIST1H2BD*, *NUDT12. TOPAZ1* and *TSNARE1*, Figure 1D). In addition, we discovered genes involved in development (*BARHL2* and *GPR101*) genes involved with cognitive ability (*TNRC6A*). Other genes near lead SNPs included *APOC1*, *ATP23*, *DTWD2*, *ECE1*, *MCHR2,* and *UXS1*. The GWAS demonstrated distinct genetic profiles between the whole cohort and male-only and female-only sub-cohort (Figure 1, A and B). After analyzing the results in FUMA [32], we detected in total three lead SNPs in the male cohort (one lead SNP, rs1252749, that was not genome-wide significant in the main analysis and was more significant in males, Supplementary Table S7), 11 lead SNPs in the female cohort (rs12581066, rs62085661, rs56270611, and rs186468751 were not genome-wide significant in the main analysis or in males, Supplementary Table S8). Finally, we performed sensitivity analyses for the number of purchases and coded non-benzodiazepines as binary trait and discovered 13 genetic loci using those with 1 or more purchases as cases. Eleven of these variants reflected nearby signals from the main analysis suggesting robustness between the quantitative and binary traits (Supplementary Figure S1, Table S9). Furthermore, using those with 2+ purchases as cases in the other binary GWAS, 10 lead SNPs reflected signals from the main quantitative analysis (Supplementary Figure S1, Table S9).

### GWAS in UKB replicates significant sleep medication purchase association of eight lead SNPs

The prescription and treatment of sleep problems differ between countries and similarly, reporting of drug use is only partially covered in biobanks that do not have harmonized data across the whole population. Therefore, to estimate the robustness of associations in a different setting we examined associations from the UK Biobank performing a similar analytical pipeline as in FinnGen. The percentages of sleep medication purchasers in UKB differed from those in FinnGen and were smaller only 4% of the cohort having sleep medication purchases (*N* = 18 421). In addition, we detected a significant overall genetic correlation between FinnGen and UKB sleep medication purchases, but the correlation was relatively low LDSC genetic correlation = 0.2, *p *= 7.4 × 10^−67^. However, the UKB GWAS shows significant association of sleep medication purchase for SNPs at alpha level 0.05 for a quarter of the loci (8 from 27 loci) including signal the canonical sleep genes *MEIS1* (rs11693221, *β* = 0.006, *p *= 0.01) and *PAX8* (rs62158170, *β* = −0.003, *p *= 0.02) and with the schizophrenia associated *TSNARE1* (rs13262595, *β* = −0.002, *p* = 0.03) and *CACNA1C* (rs2370413, *β* = 0.001, *p *= 0.04) signals but for example not with the GABRA locus. The other significant follow-up signals in UKB were for *BARHL2* (rs7527440, *β* = 0.0028, *p *= 0.005), *ECE2* (rs3914188, *β* = 0.003, *p* = 0.003), *RP11-6N13.1* (rs325485, *β* = −0.003, *p* = 0.002), and *DTWD2* (rs568201313, *β* = −0.02, *p *= 0.04) as detailed in Supplementary Table S6.

### Genetic correlation demonstrates significant shared genetic background between sleep medication purchases and psychiatric traits, sleep problems, and substance abuse traits

To understand the shared genetic background between sleep medications and psychiatric traits, sleep problems, and substance abuse, we performed genetic correlation. Out of the total of 37 trait correlations tested 30 correlated with sleep medication purchase in FinnGen (Supplementary Table S2), we found statistically significant correlation (using Bonferroni correction for multiple testing) for sleep medications against 13 out of 15 psychiatric disorder traits, 4 out of 13 sleep disorder traits, and 7 out of 10 substance abuse associated traits (Supplementary Table S2). The psychiatric traits exhibiting the most significant positive correlation with sleep medication purchases and the correlation between insomnia and sleep medications are listed in Table 1.

**Table 1.** Genetic Correlation Results Between Sleep Medications Purchases in FinnGen R7 and Psychiatric Traits

Trait	Genetic correlation	Z-score	*P*	Intercept
Depression	0.55 (0.027)	20.03	2.86E−89	−0.0071 (0.0067)
Neuroticism	0.36 (0.025)	14.46	2.10E−47	0.0059 (0.0065)
Anxiety	0.47 (0.044)	10.82	2.88E−27	0.0047 (0.0063)
Anxiety (lifetime)	0.42 (0.041)	10.15	3.16E−24	−0.0006 (0.0061)
Schizophrenia	0.25 (0.026)	9.48	2.52E−21	0.0031 (0.009)
Mood instability	0.29 (0.031)	9.32	1.18E−20	0.0107 (0.0063)
Bipolar disorder	0.31 (0.035)	8.84	9.33E−19	−0.0033 (0.0079)
ADHD	0.30 (0.043)	7.00	2.58E−12	0.0028 (0.008)
Anorexia nervosa	0.25 (0.039)	6.28	3.49E−10	0.0038 (0.0066)
PTSD	0.36 (0.072)	4.97	6.56E−07	0.0162 (0.0057)
ADHD and disruptive behavior disorder	0.27 (0.057)	4.74	2.09E−06	0.0044 (0.0062)
ASD	0.13 (0.053)	2.43	0.015	0.0108 (0.0073)
Antisocial behavior	0.14 (0.12)	1.16	0.25	0.0009 (0.0057)
Insomnia	0.44 (0.026)	16.71	1.14E−62	0.013 (0.006)

### Two-sample MR analysis suggests that anxiety, depression, insomnia, schizophrenia, and pain sensation are risk factors for sleep medication purchases

**Figure 2.**
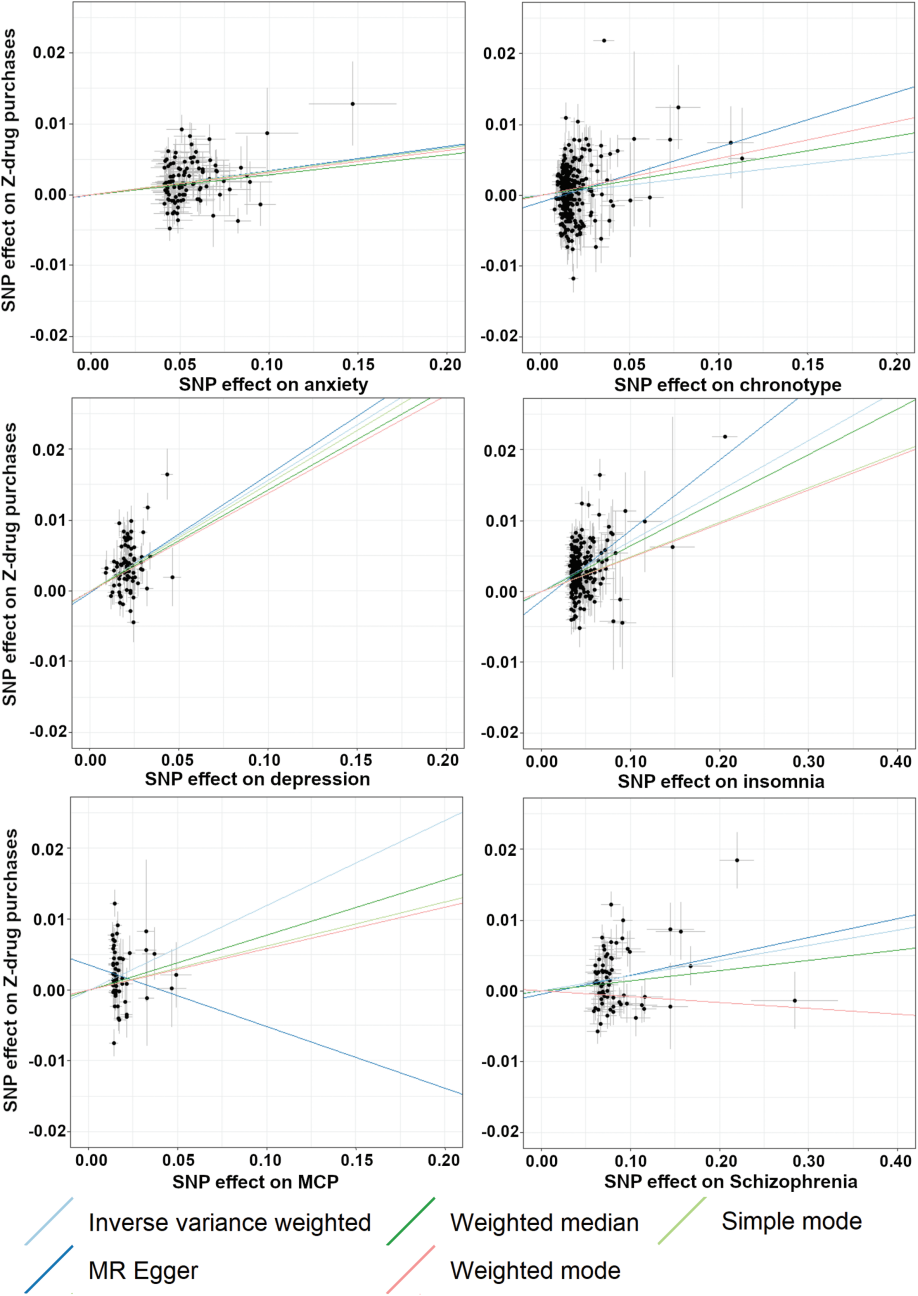
Mendelian randomization reveals causative links between sleep medication purchases and psychiatric traits. Scatter plots of MR results with sleep medication purchases as outcome against exposures/risk-factors anxiety, chronotype, depression, insomnia, multisite chronic pain (MCP), and schizophrenia. For the MCP analysis, only the weighted median and inverse-variance weighted were significant, while the negative trending MR Egger result was not significant.

We then examined the causality between the significant genetic correlation traits as risk factors for sleep medication purchases. The results from the two-sample MR analysis (Figure 2) further showed a causal relationship between the following traits as risk factors for sleep medication purchase in FinnGen; for anxiety (IVW *p *= 8.6 × 10^−12^, odds ratio (OR) [95% CI] = 1.03[1.02 to 1.04]), depression (IVW *p *= 4.5 × 10^−5^, OR[95% CI] = 2.13 [1.48 to 3.06]), insomnia (IVW *p *= 3.6 × 10^−61^, OR[95% CI] = 1.07[1.06 to 1.08]) and schizophrenia (IVW *p *= 2 × 10^−4^, OR[95% CI] = 1.02[1.01 to 1.03]). Furthermore, we could also see significant causality between multisite chronic pain as exposure and sleep medication purchases as outcome although these results were not consistent across all MR methods tested (IVW *p *= 6.5 × 10^−5^, OR[95% CI] = 1.13[1.06 to 1.2]).

### Polygenic risk score analysis highlights significant risk of decreased mood, anxiety, and insomnia with sleep medication purchases

Table 2 depicts the results of the logistic regression using generalized linear modeling of polygenic risk scores (Z-normalized, SCORE in table) of sleep medication purchases in FinnGen against psychiatric traits. We noted significant (*p *< 0.05) polygenic risk of sleep medication purchases together with the self-reported traits happiness, depressed mood, worrier/anxious feelings, insomnia, anxiety and chronotype, supporting the MR and genetic correlation results further supporting the connection between psychiatric traits and sleep medication purchases.

**Table 2.** PRS Linear Model Regression Analysis Shows Correlation Between Sleep Medication Purchases Based on FinnGen Data Against “rnorm” Sleep Medication Prescription Purchase Events in UKB and Psychiatric Traits

Model	*β*	SE	*p*	Adjusted *R*^2^
Normalized sleep medication purchases in UKB	0.014	0.0008	<2.2E−16	0.0048
Happiness (4526) ~ SCORE + covariates	0.022	0.0022	<2.2E−16	0.0086
Depressed mood (2050) ~ SCORE + covariates	0.017	0.0013	<2.2E−16	0.0091
Worrier/anxious feelings (1980) ~ SCORE + covariates	0.019	0.00094	<2.2E−16	0.024
General pain 3 + months (2956) ~ SCORE + covariates	0.010	0.0057	0.068	0.0070
Insomnia (1200) ~ SCORE + covariates	0.037	0.0012	<2.2E−16	0.032
Anxiety (21062) ~ SCORE + covariates	0.091	0.12	0.45	0.00021
Anxiety-seen doctor (2090) ~ SCORE + covariates	0.025	0.00085	<2.2E−16	0.026
Chronotype (1180) ~ SCORE + covariates	0.0063	0.0026	0.0051	0.0065
Depression (ICD10 F32,F33) ~ SCORE + covariates	0.0084	0.0004	<2.2E−16	0.003
PTSD (F43.1) ~ SCORE + covariates	0.00034	0.000051	2.33E−11	0.00052
Normalized sleep medication purchases in UKB	0.00029	0.00009	1.95E−6	0.0037
Happiness (4526) ~ covariates	−−0.0083	0.00028	<2.2E−16	0.0078
Depressed mood (2050) ~ covariates	−−0.0085	0.00017	<2.2E−16	0.0087
Worrier/anxious feelings (1980) ~ covariates	−−0.00092	0.00012	5.24E−15	0.023
General pain 3 + months (2956) ~ covariates	0.0029	0.00075	9.21E−05	0.009
Insomnia (1200) ~ covariates	0.009	0.00016	<2.2E−16	0.029
Anxiety (21062) ~ covariates	−−0.055	0.016	0.000562	0.00021
Anxiety-seen doctor (2090) ~ covariates	−−0.002	0.00011	<2.2E−16	0.023
Chronotype (1180) ~ covariates	−−0.01	0.00028	<2.2E−16	0.0064
Depression (ICD10 F32,F33) ~ covariates	−−0.00016	0.00005	0.002	0.0017
PTSD (F43.1) ~ covariates	−−6.7E−5	6.4E−6	<2.2E−16	0.00039

“SCORE” signifies Z-normalized polygenic risk score for sleep medications purchases. “Covariates” are age, sex, and genetic PCs 1–10.

### Expression quantitative trait loci analysis demonstrates significant impact on expression of genes *ECE2*, *GABRA2*, *TSNARE1*, and *CACNA1C*

While the overall observations from epidemiological analyses and genetic findings show phenotype level association between sleep medications and psychiatric traits another aspect of the findings is to understand what function the individual genetic variants may have. We therefore performed eQTL analysis with the lead variants and available tissue specific gene expression data. First, we observed significant enrichment of the eQTL signal in the brain using FUMA (Supplementary Figure S2). The GTEx platform eQTL analyses on expression in brain regions showed a significant impact on expression from several SNPs on nearby genes (Table 3). The expression of *GABRA2* and *KPNA2* demonstrated significant (based on eQTL, COLOC2, and simple sum analyses) effect on expression by their respective SNPs. *CACNA1C* demonstrated a significant negative NES in association with rs2370413 (Table 3).

**Table 3.** Bonferroni Adjusted Significant GTEx V8 eQTL Calculator Results and LocusFocus Results

Gene	SNP	*p*	NES	Tissue	COLOC2	SS
*CACNA1C*	rs2370413	0.000041	−0.38	Brain—Cerebellar Hemisphere	0.98	4.79 × 10^−8^
*GABRA2*	rs279829	2.5E−06	0.2	Brain—Cortex	0.99	2.69 × 10^−7^
*GABRA2*	rs279829	0.000056	0.18	Brain—Nucleus accumbens (basal ganglia)	0.98	5.37 × 10^−7^
*KPNA2*	rs62085661	0.000035	0.23	Brain—Caudate (basal ganglia)	0.99	4.57 × 10^−6^
*ECE2*	rs3914188	0.000051	−0.22	Brain—Cerebellar Hemisphere	0.0048	8.51 × 10^−4^
*ECE2*	rs3914188	3.8E−08	−0.42	Brain—Anterior cingulate cortex (BA24)	0.0048	6.46 × 10^−4^
*ECE2*	rs3914188	4.8E−06	−0.24	Brain—Cerebellum	0.0031	0.0028
*KIRREL3*	rs57306615	0.000048	−0.41	Brain—Spinal cord (cervical c-1)	0.0061	1.00
*TSNARE1*	rs12675715	0.000025	0.36	Brain—Spinal cord (cervical c-1)	5.6 × 10^−5^	6.03 × 10^−8^

NES signifies normalized expression score. Tissue names are based on the GTEx tissue codes. COLOC2 signifies COLOC2-based posterior probability of co-localization between GTEx V8 and GWAS data, while simple sum (SS) signifies SS probability of this co-localization. Top sections include significant co-localization based on both COLOC2 and LocusFocus SS.

## Discussion

In this paper we describe that sleep problems, primarily insomnia in the Finnish population, captured by sleep medication purchases, are strongly related to psychiatric traits at epidemiological level and at the level of individual genetic variants. Furthermore, we illustrate that the relationship between sleep medication purchases and psychiatric traits is causal so that sleep problems captured by sleep medication purchase increase the risk of psychiatric traits. Our findings indicate that sleep is connected with mental health and provides individual target genes to explore further.

Our GWAS analysis of FinnGen R7 produced 27 independent loci and 11 loci were replicated in UKB, a dataset with substantially different frequencies of sleep medication purchases implicating a robust signal for the discovered variants. Furthermore, we confirmed a significant correlation of sleep medication purchases and the frequency of psychiatric traits (*β* = 1.35, *p *< 2 × 10^−16^). It is worth noting that the indication of prescribing sleep medications (non-benzodiazepine drugs) in Finland is insomnia. These findings therefore connect the clinical use of drugs with the earlier implicated role of insomnia and sleep problems in psychiatric traits.

In agreement with the variant level results, our genetic correlation analysis demonstrated significant correlation between sleep medication purchases and 13 out of 17 psychiatric disorder traits, 4 out of 13 sleep disorder traits, and 7 out of 10 substance abuse associated traits. The genetic correlation between sleep medication purchases in FinnGen compared to UKB had a highly significant (7.4 × 10^−67^) but relatively low overall correlation of 0.2. This could suggest that although there are significant genetic similarities in uses of sleep medications, there may also be discrepancies in policies and culture of sleep medication purchases between countries that result in an effect on the GWAS. Furthermore, there is a distinct difference in FinnGen and UKB cohorts in terms of cohort incidences of psychiatric traits, and the overall higher age of FinnGen (Supplementary Tables S3–S5).

Using two-sample MR we detected a statistically significant relationship with anxiety, depression, insomnia, multisite chronic pain, and schizophrenia as risk factors for the outcome of being prescribed sleep drugs. Furthermore, the causative relationship between pain and sleep problems such as insomnia has been demonstrated in previous studies [33] accentuating the connection between sleep and psychiatric traits and connecting our work with earlier literature of insomnia. Our work demonstrates that sleep problems have a clinical impact as an outcome of psychiatric traits.

Insomnia commonly affects individuals with schizophrenia [34]. Based on the GWAS results, we detected two SNPs; rs12675715 and rs13262595 that are located in *TSNARE1*, a gene that encodes the tSNARE1 (t-SNARE containing domain 1) protein, which is associated with schizophrenia onset and bipolar disorder [35, 36]. *TSNARE1* has recently been reported to be involved in intracellular transport in cortical neurons [36, 37]. Using publicly available datasets from the UKB and GWAS catalog, we noted that rs13262595 had significant positive associations with traits such as intelligence (*p *= 9.1 × 10^−10^) and cognitive performance (*p *= 1.3 × 10^−11^), and significant negative association with worry (*p *= 3.2 × 10^−13^), feeling tense (*p *= 1.3 × 10^−9^), and nervous feelings (*p *= 9.7 × 10^−10^). It has also been identified as a lead SNP in a GWAS for the mental health questionnaire completion phenotype in the UKB [38].

The SNP rs11639856 was found in the exonic region of *TNRC6A* (CADD score = 15.1), which encodes a nucleus-cytoplasm transport protein [39]. The SNP was found to be significantly negatively associated with weight (*p *= 1.6 × 10^−15^) and body mass index (*p *= 1.2 × 10^−8^) in UKB. Furthermore, we also detected the intronic lead SNP rs2370413 in *CACNA1C*. The *CACNA1C* gene encodes the α subunit of a voltage-dependent calcium channel [40]. Mutations in the gene have been associated with schizophrenia, bipolar disorder, and autism [40]. Finally, rs2370413 has demonstrated significant positive association with traits such as schizophrenia vs major depressive disorder (*p *= 6.9 × 10^−14^) and schizophrenia vs autism spectrum disorder (*p *= 2.5 × 10^−14^). These findings highlight the connection between sleep problems and schizophrenia.

Additionally, we also detected one lead SNP (rs279829) in *GABRA2*. The *GABRA2* gene encodes the α2 subunit of α-containing GABA_A_ receptors, which have been linked to mood disorders and CNS diseases such as Alzheimer [41]. Furthermore, GABA_A_ is one of the targets of sleep medications. This finding implicates a possible direct role for treatment targeting the relevant disease pathway. In agreement, the GABRA_A_ receptor has been demonstrated to be associated with the development of alcohol dependence, mood, and epilepsy [42, 43] linking the genetic association with sleep medication with related comorbid and severe diseases. Our UKB replication further highlights SNPs associated with the genes *CACNA1C*, *PAX8*, *MEIS1*, *ECE2*, and *TSNARE1*. However, the overall low SNP significance overlap (Supplementary Table S6) and low genetic correlation score suggests that sleep medications in the UKB and in FinnGen may capture different traits (LDSC genetic correlation = 0.2, *p *= 7.4 × 10^−67^). The overall result indicates a shared genetic etiology of *CACNA1C*, *PAX8*, *MEIS1*, *ECE2*, and *TSNARE1* even with possible differences in prescription practices.

The PRS analysis coupled with the MR suggests a polygenic nature behind the sleep medication purchases in FinnGen that is linked to mood, depression, and mood-altering psychiatric traits. This corresponds to previous MR studies describing significant causality between insomnia and greater depressive symptoms, schizophrenia, ADHD, neuroticism, and anxiety [16, 17].

The sensitivity test GWAS using a binary phenotype (1+ or 2+ purchases as cases) further provides robustness to the overall results. In particular, both of the sensitivity GWASs overlapped (within 1 million bp) with the quantitative analysis for the genes *MEIS1*, *PAX8*, *TOPAZ1*, and *TSNARE1*. The 1+ purchase GWAS also overlapped with the *KIRREL3* and *CANCA1C* loci. The 2+ purchase GWAS overlapped with *BARHL2*, *ECE2*, and *GPR101*.

Here, we have presented GWAS, genetic correlation, MR, eQTL, co-localization, and PRS data using the Finnish FinnGen cohort, showing that sleep medication purchases are significantly linked with anxiety, depression, chronic pain, insomnia, and schizophrenia. Thus, this investigation serves as a foundation for further clinical studies in the interplay between sleep management and psychiatric traits. We report patient groups that may benefit significantly from sleep care. Furthermore, we validated GWAS data with the UKB cohort as well, but due to the differences in the cohort recruitment and composition, a straight comparison is difficult. This may point out the strengths of having a relatively homogenous cohort such as the FinnGen project to study these types of traits influenced by national policies. For example, one study covering the years 2008–2011 noted a significant reduction in non-benzodiazepine (and benzodiazepine) sales in Finland while much smaller in the United Kingdom, based on different national drug use policy campaigns [19]. We also note that there may be significant differences in the reasons for prescription of non-benzodiazepine drugs; the cause may be temporary due to life events, or chronic due to psychiatric disorders. While these differences make comparisons difficult, it may be that overlapping results show robustness due to the diversity of the cohorts. Another potential limitation is the focus of our study on non-benzodiazepine drugs. An extension using other drugs for sleep treatment could provide additional power to the study, although using non-benzodiazepine drugs allow for the focused capture of insomnia related traits. On the gene-level the results highlight important genetic mechanisms through *TSNARE1*, *GABRA2*, *ECE2*, *PAX8*, *MEIS1*, and *CACNA1C.* The results validate variation in canonical sleep disorder genes such as *MEIS1*, or *PAX8* which have been reported as significant in insomnia GWAS [16, 17], as increasing sleep medication purchase. These findings indicate that better diagnostics of the underlying diseases may benefit correct treatment. Overall, our study indicates that understanding the link between improving sleep and beneficial effects on patient levels of mood issues, anxiety, insomnia, schizophrenia, and depression is important and may aid patient care. This has been explored in recent randomized control trials [2] and we believe this is an important avenue moving forward.

## Supplementary Material

zsad279_suppl_Supplementary_Tables_S1-S9_Figures_S1-S2

## Data Availability

The genome-wide summary statistics used in this article can be accessed through the GWAS catalog.
